# Lipid-Lowering Therapy after Acute Coronary Syndrome in Outpatient Practice—How to Achieve Goal

**DOI:** 10.3390/jcm12206579

**Published:** 2023-10-17

**Authors:** Renata Rajtar-Salwa, Beata Bobrowska, Jakub Batko, Stanisław Bartuś, Paweł Petkow-Dimitrow, Agata Krawczyk-Ożóg

**Affiliations:** 1 Department of Cardiology and Cardiovascular Interventions, University Hospital, 30-688 Kraków, Poland; rrajtar@su.krakow.pl (R.R.-S.); bobrowska.beata@gmail.com (B.B.); mbbartus@cyfronet.pl (S.B.); dimitrow@mp.pl (P.P.-D.); 2 Department of Anatomy, Jagiellonian University Medical College, 31-008 Kraków, Poland; kubabatko5@gmail.com; 3 CAROL—Cardiothoracic Anatomy Research Operative Lab, Department of Cardiovascular Surgery and Transplantology Institute of Cardiology, Jagiellonian University Medical College, 31-008 Krakow, Poland; 4 2nd Department of Cardiology, Jagiellonian University Medical College, 30-688 Kraków, Poland

**Keywords:** acute coronary syndromes, hypercholesterolemia, secondary prevention, LDL-C

## Abstract

Background: Secondary prevention of cardiovascular disease involves the use of optimal pharmacological treatment and modification of risk factors through lifestyle changes. Recent evidence demonstrates that the major initiating event in atherogenesis is the storage of low-density lipoproteins. Objectives: We aimed to compare the efficacy in achieving the therapeutic lipid target in relation to the frequency of follow-up at selected time points and to determine the safety and tolerability of cholesterol-lowering drugs (statins, ezetimibe). Methods: This was a prospective analysis of 72 consecutive patients hospitalized for acute coronary syndrome: ST-elevation myocardial infarction (STEMI) and non-ST-elevation myocardial infarction (NSTEMI). Patients were consecutively divided into two groups: first, with follow-up and laboratory tests at 1, 3, 6 and 12 months after hospital discharge, including 32 patients; second, including 40 patients with follow-up and laboratory tests 12 months after hospital discharge. Results: A significant reduction in LDL-C level was observed at 12 months in both groups. LDL-C level was significantly lower in group 1 than in group 2 after 12 months (*p* = 0.02). Total cholesterol level was significantly lower in group 1 than in group 2 after 12 months. After 12 months of therapy, 21 (65.6%) patients in group 1 and 17 (42.5%) in group 2 had LDL-C < 1.4 mmol/L. In group 1, we observed a significant decrease in LDL-C, triglyceride, and total cholesterol levels at 1, 3, 6 and 12 months (*p* < 0.05). Conclusions: The group of patients with more frequent follow-up visits showed a greater reduction in LDL-C level than the group with only one visit after a 12-month hospital discharge.

## 1. Introduction

Coronary artery disease is a major cause of morbidity, mortality and disability worldwide. Acute coronary syndrome, as a spectrum of symptoms, can be divided into two main groups: unstable angina and acute myocardial infarction, which can be differentiated by the cardiac troponin elevation and changes, with at least one value over the 99th percentile [1]. Myocardial infarction can be further divided into ST-elevated myocardial infarction (STEMI) or non-ST-elevated myocardial infarction (NSTEMI) based on electrocardiographic findings (elevation of the ST segment) [1]. Determining and controlling optimal treatment for patients with this disease is a crucial clinical problem. People diagnosed with cardiovascular disease are at significantly increased risk for adverse events such as myocardial infarction, stroke and even death. Patients with atherosclerotic cardiovascular disease, who suffered from a second vascular event within two years despite implementation of maximally tolerated statin-based therapy, are in greater risk of its recurrence. Therefore, an LDL-C goal of <1.0 mmol/L may be considered as a therapeutic target [2]. These individuals are a top clinical priority when implementing secondary prevention interventions [3]. Prevention is characterized as a coordinated set of interventions, whether at the population level or on an individual basis, aimed at eliminating or mitigating the consequences of cardiovascular disease and its associated impairments. Secondary prevention of cardiovascular disease involves the use of optimal pharmacological treatment and modification of risk factors through lifestyle modifications including increased physical activity, optimal diet, and cessation of unhealthy habits such as smoking [4]. A growing number of patients survive their initial cardiovascular events and remain vulnerable to potential recurrence. Recent evidence demonstrates that the primary trigger of atherogenesis is the incorporation of low-density lipoprotein (LDL-C) and other high-cholesterol lipoproteins into the arterial wall. The indisputable causal involvement of LDL-C and other apo-B-containing lipoproteins in the progression of coronary artery disease has been convincingly demonstrated by genetic, observational and interventional studies [5]. Continued lowering of LDL-C levels results in an additional and confirmed reduction in the incidence of myocardial infarction, revascularization and ischemic stroke, with each 1.0 mmol/L reduction reducing the annual rate of these serious vascular events by slightly more than 20% [6]. Prospective studies, randomized trials and Mendelian randomization studies have collectively demonstrated that elevated LDL-C serves as a contributing factor to cardiovascular disease. Across a spectrum of LDL-C concentrations, the principle of “lower is better” holds true, without a discernible lower limit, at least until reaching 1 mmol/L [7]. Current European Society of Cardiology guidelines recommend lipid-lowering treatment in secondary prevention, with the ultimate goal of achieving an LDL-C level below 1.4 mmol/L (55 mg/dL) and a reduction of more than 50% from the baseline. In addition, for secondary prevention, it is recommended to maintain apolipoprotein B levels below 0.65 g/L [7,8]. Many patients do not achieve this therapeutic goal. The prevalence of an unhealthy lifestyle remains substantial, and cardiovascular disease risk factors are often inadequately addressed, even in patients who are classified as being at high (residual) risk for cardiovascular disease [9]. Patients with acute coronary syndrome require continuous cardiac monitoring as a high-risk group for recurrent cardiovascular events. Adoption of healthier lifestyle habits by patients is best achieved through structured prevention initiatives, likely because of the thorough monitoring and diverse expertise they provide [10]. However, in routine clinical practice, both patients and health care providers encounter difficulties in ensuring adherence to healthy lifestyle habits and prescribed medications.

### Aims

The aims of this study were to compare efficacy in achieving the therapeutic lipid target as a function of the frequency of follow-up visits at selected time points and to determine the safety and tolerability of cholesterol-lowering drugs (statins, ezetimibe).

## 2. Materials and Methods

### 2.1. Study Population

The study group included 72 consecutive patients hospitalized for acute coronary syndrome (STEMI or NSTEMI) in the Clinical Department of Cardiology and Cardiovascular Interventions, University Hospital in Krakow, Poland from June 2021 to June 2022. Exclusion criteria included: inability to obtain informed consent from the patient to participate in the study, inability of the patient to travel for follow-up, patients with a prognosis of less than 1 year survival, patients without diagnosis of myocardial infarction (STEMI or NSTEMI) and patients with neurological disorders that interfere with obtaining medical history. Patients were consecutively divided into two groups, based on random number generated during collection of patient informed consent:

Group 1:  Patients will be invited for follow-up examinations and laboratory tests 1, 3, 6 and 12 months after hospital discharge.
Group 2:  Patients will be invited for follow-up examinations and laboratory tests 12 months after hospital discharge.

### 2.2. Data Collection

Demographic and medical history data were collected from all patients. The presence of concomitant diseases, including cardiac and peripheral interventions, atrial fibrillation, arterial hypertension, diabetes mellitus, peptic ulcer disease, thyroid disease, previous cerebrovascular accidents, chronic kidney disease, active smoking, and lipid-lowering therapy before admission, was recorded. Data were also collected on left ventricular ejection fraction during hospitalization and the type of pharmacotherapy the patient was taking on the day of hospital discharge.

Fasting blood samples were obtained at patient admission and at follow-up visits. At baseline, levels of LDL-C, total cholesterol, high-density lipoprotein (HDL-C), triglycerides, alanine transaminase, aspartate transaminase, thyroid-stimulating hormone, creatine kinase, apolipoprotein B, lipoprotein (a), fasting glucose (in patients without diabetes mellitus or glycated hemoglobin (HbA1c) in diabetic patients), and creatinine were measured.

Follow-up and all laboratory tests, except apolipoprotein B and lipoprotein (a), were performed at follow-up visits after 12 months in all patients. Patients in group 1 also had follow-up examinations and lipid profile tests at 1, 3 and 6 months. The researchers had no effect on the medications given to patients at hospital discharge after myocardial infarctions. A change in lipid-lowering treatment based on the test results was made only at follow-up. ”Very brief advice” for smoking cessation (Ask, Advice, Act) were performed in each visit with active smoking patients. Patients were recommended to perform the following simple dietary modifications: increase daily vegetable intake to five servings a day, eat a variety of protein food with no more than three servings of red meat a week and exclusion of highly processed meat, reduce use of saturated fat and processed meals, replace salt with herbs and spices for flavoring.

### 2.3. Statistical Analysis

Patients were divided into two subgroups on the basis of their follow-up rate, as previously defined. The collected data were tested for normality using the Shapiro–Wilk test, tabulated, and reported as means with standard deviations (mean ± SD). The Mann–Whitney U test was used to compare previously defined groups at baseline and 12-month follow-up. The Wilcoxon test was used to analyze differences between blood sample results at baseline and at 1 month, 3 months, 6 months and 12 months in each subgroup separately. Statistical analyses were performed using IBM SPSS Statistics for Windows, version 29.0 (IBM Corp., Armonk, NY, USA). A *p*-value of less than 0.05 was considered statistically significant.

## 3. Results

The mean age of the included patients was 63.4 ± 12.5 years. Men accounted for 63.5% (47 patients) of the study group. The clinical and demographic characteristics of the patients in both groups are shown in Table 1.

The percentage reduction in LDL-C level after 12 months was 47 ± 23% in group 1 and 26 ± 46% in group 2. A significant reduction in LDL-C level was observed after 12 months in both group 1 (2.64 ± 1.28 vs. 1.21 ± 0.54 mmol/L; *p* < 0.001) and group 2 (2.86 ± 1.24 vs. 1.84 ± 1.17 mmol/L; *p* < 0.001) compared to baseline. LDL-C level was significantly lower in group 1 than in group 2 after 12 months (*p* = 0.02). The percentage reduction in total cholesterol level at 12 months was 28 ± 18% in group 1 and 12 ± 36% in group 2. A significant reduction in total cholesterol level from the baseline was observed at 12 months in both group 1 (4.60 ± 1.40 vs. 3.10 ± 0.60 mmol/L; *p* = 0.001) and group 2 (4.90 ± 1.60 vs. 4.01 ± 1.40 mmol/L; *p* = 0.002). Total cholesterol levels were significantly lower in group 1 than in group 2 after 12 months (*p* = 0.001) (Table 1). We did not observe significant increases in alanine transaminase, aspartate transaminase, thyroid-stimulating hormone, creatinine, and creatine kinase. On the contrary, some of these values were even lower than at admission, including aspartate transaminase (*p* = 0.020), thyroid stimulating hormone (*p* = 0.030) and creatine kinase (*p* = 0.003) in group 1 and alanine (*p* = 0.002) and aspartate transaminase (*p* = 0.002), and creatine kinase (*p* = 0.005) in group 2. In addition, we did not observe any cardiovascular events throughout the follow-up period. Detailed data on all blood test results can be found in Table 2.

In group 1, we observed a significant decrease in LDL-C, triglyceride and total cholesterol levels at 1-month follow-up and for LDL at 3-month follow-up (*p* < 0.05). The statistically significant decrease was stabilized at 6-month and 12-month follow-up visits. The detailed results are shown in Table 3.

In our entire study group, 23 (31.9%) patients received a maximum statin dose (fourteen taking 40 mg rosuvastatin and nine taking 80 mg atorvastatin). Only seven patients were prescribed ezetimibe at the time of discharge (two of whom had taken it previously). In group 1, the statin dose was increased in 13 patients, and another 11 patients were also prescribed ezetimibe during the follow-up period. Detailed statin dosage at 12-month follow-up visit can be found in Table 4.

Eighteen (25.0%) of the patients (eleven (34.4%) in group 1 and seven (17.5%) in group 2) admitted to being current smokers, and nine (12.5%) of all subjects were ex-smokers. Encouragingly, eight patients in group 1 decided to quit smoking during the observation period (*p* = 0.021), while only three subjects in group 2 quit smoking (*p* = 0.38).

## 4. Discussion

The analysis shows that both study groups achieved a significant decrease in LDL-C and total cholesterol levels after a 12-month follow-up period. After 12 months of therapy, 21 (65.6%) patients in group 1 and 17 (42.5%) in group 2 had LDL-C < 1.4 mmol/L, as recommended by the guidelines. However, the group with more frequent follow-up visits showed a greater reduction than the group with only one visit after a 12-month hospital discharge. In group 1, patients had the opportunity to see a cardiologist more frequently and thus receive modified lipid-lowering therapy to achieve LDL-C < 1.4 mmol/L (<55 mg%). These individuals were also motivated to take their medications consistently, follow a specific diet and quit smoking.

Patients who have suffered a myocardial infarction are at increased risk of recurrence of cardiovascular events. In such cases, lipid control should be considered as part of a holistic strategy for global risk reduction. This strategy includes lifestyle adjustments, risk factor management and the use of pharmaceutical approaches. Despite the recognized benefits of lowering LDL-C levels in patients after myocardial infarction, achieving desired LDL-C targets in this high-risk setting remains suboptimal [4,11,12].

The guidelines suggest a stepwise approach to intensify treatment to achieve goals, including for LDL-C levels. However, in patients at very high risk, it is worth considering a more aggressive approach and moving directly toward the low LDL-C target [8]. Based on the most recent available data, high-intensity statin treatment is recommended within the first 1 to 4 days after hospitalization for acute coronary syndrome [7]. If the targeted goals cannot be achieved with the highest tolerated dosage of a statin alone, it is recommended that a combination with ezetimibe [13] and the next proprotein convertase subtilisin/kexin type 9 inhibitors be considered [14,15]. Based on regular physician visits and lipid profile monitoring in group 1, modification of lipid-lowering therapy (increasing statin dosage or adding ezetimibe) was performed to achieve LDL-C goals. In our group of patients, none of the participants received proprotein convertase subtilisin/kexin type 9 inhibitors because of the high cost and restrictive criteria for inclusion in the National Health Fund drug program in Poland [16].

Compelling evidence from pathophysiological, observational and genetic studies suggests a possible causal relationship between lipoprotein (a) levels and the development of atherosclerotic cardiovascular disease [17], although this risk factor appears to exert a much milder influence on the majority of people compared with LDL-C [16,17]. People with extremely high lipoprotein (a) levels of more than 1.80 g/L (430 nmol/L) may have an increased lifetime risk of cardiovascular disease, comparable to people with heterozygous familial hypercholesterolemia [18,19]. Given that approximately 90% of a person’s lipoprotein (a) level is inherited, markedly elevated lipoprotein (a) levels could potentially indicate a novel inherited lipid disorder associated with markedly increased lifetime cardiovascular risk that is twice as common as heterozygous familial hypercholesterolemia [9]. In our study group, two patients had lipoprotein levels greater than 1.8 g/L, and they were men with their first myocardial infarction who were over 60 years of age.

One of the most important lifestyle factors was tobacco use. Abstaining from smoking is associated with a substantial reduction in all-cause mortality risk in patients diagnosed with coronary artery disease [20]. More than one-third of our cohort were active smokers or had a history of smoking. The “Very brief advice” on smoking is a proven 30-s clinical intervention that identifies smokers, advises them on the best method for quitting, and supports subsequent quit attempts [8]. It is an evidence-based intervention that involves three steps: asking patients about their tobacco use, advising them that the best method for quitting is a combination of medication and behavioral support, and taking action by supporting them in a quit attempt using available cessation aids [21]. Eleven of the active smokers (eight individuals in group 1 and three individuals in group 2) quit smoking after myocardial infarction. Continued education of patients after myocardial infarction is an effective method for smoking cessation [22].

It is worth noting that in our study group, more than a quarter (20 patients) of our patients were already on secondary prevention. At baseline, LDL levels were above 1.4 in 70% of them, suggesting that these patients were noncompliant individuals. If these patients do not receive regular medical monitoring, their risk of cardiovascular disease recurrence will never decrease.

The Comprehensive Care after Myocardial Infarction program in Poland that provides care and support to patients after an acute coronary syndrome. The main goal of this program is to improve patient outcomes and reduce the risk of future cardiac events. It includes several key components. First, it emphasizes regular physician follow-up and close monitoring of the patient’s condition. Second, the program focuses on lifestyle changes, such as a healthy diet, regular exercise and quitting smoking. In addition, patients are educated about the importance of taking prescribed medications consistently and as prescribed, and psychological support is provided [23]. The most common practice is to perform a lipid profile after 12 months of therapy. However, in our observation, this should be performed much earlier. In group 1, the largest changes were observed at the 1-month follow-up visit, which emphasize its importance in reaching the therapeutic point in patients after myocardial infarction. Following 3-month and 6-month follow-up visits were important for maintaining the appropriate levels of lipoproteins and correction of the lipid-lowering therapy. However, the main limitation of the proposed strategy is a need for increased capacity of cardiologists which is associated with additional costs. Implementation of the 1-month follow-up visit during cardiological rehabilitation may decrease the need for three additional visits to two: 3-month and 6-month follow-up. Interventions during those visits can be performed by general practitioners or family doctors, which additionally may increase the effectiveness of the therapy because of the specific doctor–patient relationship in relations with family doctors. The proposed extension of the medical specialists included in the patients’ follow-up may be helpful to decrease the impact of the previously mentioned limitation. Additionally, potential benefits from the prevention of cardiovascular events may lower the general cost of hospitalization and care associated with each patient.

Statin-associated muscle symptoms have been reported in 10–15% of subjects in observational studies [7]. They are not as common in blinded randomized trials of statins versus placebo [24,25]. Available information on the appropriate tests to monitor lipid levels in patients undergoing treatment is limited. The same limited data apply to tests assessing potential toxicity, such as alanine transaminase and creatine kinase [7]. Recent analyzes provide positive evidence for the safety of prolonged statin therapy, and it is noted that cases of statin-induced liver injury are extremely rare [26]. In our study group, we did not observe any increase in alanine transaminase, aspartate transaminase or creatine kinase during the observation period. In addition, patients did not report musculoskeletal pain. Additionally, we observed a statistically significant decrease in the level of creatine kinase comparing the baseline and 12-month follow-up visit results, which may be associated with high creatine kinase levels after myocardial infarction. A decrease in aspartate transaminase was statistically significant in both study groups after 12-month follow-up and was within the correct ranges; however, the alanine transaminase significantly decreased only in the control group. However, its levels observed within group 1 should not be associated with potential hepatologic complications, due to its slight elevation without aspartate transaminase elevation. Thyroid-stimulating hormone changes in group 1 should be interpreted with caution as both results are within the normal range.

In our study, we would like to emphasize how important such an approach is for the group of patients with acute coronary syndrome. Only regular medical follow-up and monitoring of lipid levels can achieve the therapeutic goals. The impact of consistent lipid monitoring on encouraging patients to adhere to lifestyle changes or medication routines that positively impact their health has been highlighted in several research papers. It remains uncertain whether the monitoring process alone is critical to achieving this outcome or whether a mix of education, regular communication and assessment of adherence is required.

The main limitation of the study is the small sample size, which is due to the fact that many patients were not willing to participate in the follow-up visits to our department. Additionally, there were no patients with unstable angina included in the study, as they were excluded according to the study protocol. However, they should be considered as a potential additional patient cohort, for whom it should be assessed whether analogous intervention may be beneficial. Additionally, this is a prospective, non-randomized study, which should be noted when interpreting the study results. The main reasons to not classify this study as a randomized controlled trial are as follows: the large risk of a staff member identifying a patient’s group allocation during the study period and limited number of staff on the study. These limitations impeded the staff members’ possible blinding according to the specific character of the study (connected with different patterns of follow-up visits) and led the researchers to decide at the time of writing the study protocol to perform this study as a prospective study with as many randomization factors as possible. Nevertheless, we remain confident that our study will help to raise clinical awareness of the importance of regular patient monitoring after acute coronary syndrome.

In the future, research including additional patient groups should be considered to determine if the frequency of follow-up visits impact the effectiveness of the new lipid-lowering therapies. For example, implementation of bempedoic acid in patients with statin intolerance may put a new light on achieving the lipid-lowering therapeutic target with this drug and perform detailed comparison with statins in short-term and long-term effects of the therapy. It was proven that bempedoic acid, compared to placebo, decreased the risk of major adverse cardiovascular events, which is comparable to the effect of maximally tolerated statin therapy [27]. Additionally, use of inclisiran may be considered, which was proven to significantly decrease the levels of low-density lipoprotein compared to placebo, with a low rate of adverse effects. It may be especially useful in a cohort of patients with statin intolerance. Additionally, its dosage, comfortable for the patient, may be connected with follow-up visits [28].

## 5. Conclusions

The group of patients with more frequent follow-up visits had a greater reduction compared to the group with only one visit at 12-month hospital discharge. At 12 months, 65.6% of patients in group 1 and 42.5% of patients in group 2 achieved LDL-C levels below 1.4 mmol/L, as recommended by the guidelines. No increase in creatine kinase, alanine transaminase, aspartate transaminase and creatinine were observed during escalation of lipid-lowering therapy.

## Figures and Tables

**Table 1:** Clinical and demographic characteristics of patients. *—percentages calculated based on number of current smokers at time of hospitalization discharge (eleven in group 1, seven in group 2). Statistically significant *p*-values bolded.

	Group 1 (*n* = 32)	Group 2 (*n* = 40)	*p*-Value	All (*n* = 72)
Age, years, mean (SD)	61.9 ± 11.7	64.5 ± 13.1	0.31	63.4 ± 12.5
Male, *n* (%)	25 (78.1%)	22 (55.0%)	0.049	47 (63.5%)
BMI, mean (SD)	29.6 ± 4.5	28.5 ± 4.9	0.26	29.0 ± 4.7
Previous myocardial infarction, *n* (%)	8 (25.0%)	12 (30.0%)	0.79	20 (27.8%)
Previous percutaneous coronary intervention, *n* (%)	5 (15.6%)	8 (20.0%)	0.76	13 (18.1%)
Previous coronary artery bypass grafting, *n* (%)	2 (6.3%)	1 (2.5%)	0.58	3 (4.2%)
Hypertension, *n* (%)	15 (46.9%)	31 (77.5%)	**0.01**	46 (63.9%)
Hypercholesterolemia, *n* (%)	11 (34.4%)	14 (35.0%)	1.00	25 (34.7%)
Diabetes mellitus type II, *n* (%)	9 (29.0%)	11 (27.5%)	1.00	20 (28.2%)
Atrial fibrillation, *n* (%)	3 (9.4%)	5 (12.5%)	1.00	8 (11.1%)
Chronic obstructive pulmonary disease, *n* (%)	1 (3.1%)	1 (2.5%)	1.00	2 (2.8%)
Thyroid disorders, *n* (%)	5 (15.6%)	5 (12.5%)	0.74	10 (13.9%)
Chronic kidney disease, *n* (%)	6 (18.8%)	2 (5%)	0.13	8 (11.1%)
Pulmonic disease, *n* (%)	0 (0%)	4 (10%)	0.13	4 (5.6%)
Previous transient ischemic attack and/or stroke, *n* (%)	3 (9.4%)	2 (5%)	0.58	5 (6.9%)
Presence of implantable pacemaker, *n* (%)	1 (3.1%)	0 (0%)	0.44	1 (1.4%)
Presence of cardiac resynchronization therapy device/cardioverter defibrillator, *n* (%)	0 (0%)	0 (0%)	-	0 (0%)
Ever smoker, *n* (%)	15 (46.9%)	12 (30.0%)	0.19	27 (37.5%)
Left ventricular ejection fraction (%), mean (SD)	49.5 ± 11.1	47.1 ± 9.2	0.21	48.2 ± 10.1
Statins before hospitalization, *n* (%)	Rosuvastatin	17 (53.1%)	18 (45%)	0.64
	Atorvastatin	15 (46.9%)	22 (55%)	0.64
Discharge medications				
Beta blocker, *n* (%)	29 (90.6%)	36 (90%)	1.00	65 (90.3%)
Calcium channel blocker, *n* (%)	28 (87.5%)	35 (87.5%)	1.00	63 (87.5%)
Angiotensin-converting enzyme inhibitors, *n* (%)	25 (78.1%)	27 (67.5%)	0.43	52 (72.2%)
Angiotensin receptor blockers, *n* (%)	0 (0.0%)	4 (10%)	0.12	4 11(5.6%)
Diuretics, *n* (%)	18 (56.3%)	24 (60%)	0.81	42 (58.3%)
Ezetimibe, *n* (%)	5 (15.6%)	2 (5%)	0.23	7 (9.7%)
Clopidogrel, *n* (%)	7 (21.9%)	12 (30%)	0.59	19 (26.4%)
Prasugrel, *n* (%)	0 (0%)	0 (0%)	-	0 (0%)
Ticagrelor, *n* (%)	24 (75%)	26 (65%)	0.44	50 (69.4%)
Acetylsalicylic acid, *n* (%)	32 (100%)	40 (100%)		72 (100%)
Anticoagulant therapy, *n* (%)	4 (12.5%)	4 (10%)	1.00	8 (11.1%)
Apolipoprotein B (g/L), mean (SD)	0.65 ± 0.17	0.76 ± 0.32	0.25	0.71 ± 0.27
Lipoprotein (a) (g/L), median (Q1;Q3)	0.37 (0.06;1.00)	0.17 (0.07;0.96)	0.68	0.19 (0.06;0.96)
Maximal statin dosage at 12-month follow-up visit, *n* (%)	Rosuvastatin	8 (25%)	6 (15%)	0.29
	Atorvastatin	4 (12.5%)	5 (12.5%)	1.00
Ezetimibe at 12-month follow-up visit *n* (%)	11 (34,4%)	7 (17.5%)	0.10	18 (25%)
Smoking cessation at 12-month follow-up visit * *n* (%)	8 (72.7%)	3 (42.9%)	0.21	11 (61.1%)

**Table 2:** Results of blood laboratory tests reported as mean ± standard deviation at baseline and during follow-up in both group of patients. LDL—low-density lipoprotein; HDL—high density lipoprotein. Statistically significant *p*-values bolded.

	Baseline			12 Months			Baseline vs. 12 Months (*p*-Value)	
	Group 1	Group 2	*p*-Value 1 vs. 2	Group 1	Group 2	*p*-Value 1 vs. 2	Group 1	Group 2
Total cholesterol (mmol/L)	4.60 ± 1.40	4.90 ± 1.60	0.490	3.10 ± 0.60	4.01 ± 1.40	**0.001**	**0.001**	**0.002**
LDL (mmol/L)	2.64 ± 1.28	2.86 ± 1.24	0.530	1.21 ± 0.54	1.84 ± 1.17	**0.020**	**<0.001**	**<0.001**
HDL (mmol/L)	1.21 ± 0.38	1.23 ± 0.28	0.620	1.29 ± 0.36	1.37 ± 0.31	0.260	0.098	**0.007**
Triglyceride (mmol/L)	1.67 ± 0.82	1.89 ± 1.11	0.450	1.38 ± 0.77	1.73 ± 0.79	**0.020**	**0.017**	0.670
Alanine transaminase (U/I)	43.90 ± 32.95	40.86 ± 34.88	0.790	35.00 ± 16.83	24.57 ± 9.19	**0.020**	0.960	**0.002**
Aspartate transaminase (U/I)	59.10 ± 50.09	66.53 ± 51.49	0.630	31.64 ± 11.7	26.91 ± 8.06	0.110	**0.020**	**0.002**
Thyroid-stimulating hormone (uIU/mL)	2.17 ± 1.42	1.79 ± 1.70	0.150	1.46 ± 0.91	1.80 ± 1.29	0.410	**0.030**	0.860
Creatine kinase (U/I)	488 ± 588	329 ± 294	0.560	143 ± 112	120 ± 63	0.760	**0.003**	**0.005**
Creatinine (umol/L)	122.7 ± 122.5	92.9 ± 38.1	0.650	129.8 ± 120.9	97.8 ± 46.7	0.480	0.120	0.260
Glucose (in patients without diabetes mellitus) (mmol/L)	6.73 ± 2.17	6.82 ± 2.27	0.950	6.76 ± 3.89	6.98 ± 3.8	0.640	0.290	0.360
Glycated hemoglobin (diabetes) (%)	7.90 ± 1.60	7.60 ± 1.70	1.000	6.90 ± 0.80	7.20 ± 1.30	0.890	0.140	0.410

**Table 3:** Results of blood laboratory tests reported as mean ± standard deviation at baseline, 1, 3, 6 and 12 months of follow-up in group 1. LDL-C—low-density lipoprotein; HDL—high density lipoprotein. Statistically significant *p*-values bolded.

	Baseline	1 Month	*p*-Value Baseline vs. 1 Month	3 Months	*p*-Value 1 Month vs. 3 Months	6 Months	*p*-Value 3 Months vs. 6 Months	12 Months	*p*-Value 6 Months vs. 12 Months
Total cholesterol (mmol/L)	4.60 ± 1.40	3.30 ± 0.80	**0.014**	3.10 ± 0.60	0.071	3.20 ± 0.90	0.782	3.10 ± 0.60	0.345
LDL (mmol/L)	2.64 ± 1.28	1.50 ± 0.70	**0.016**	1.22 ± 0.43	**0.011**	1.31 ± 0.58	0.530	1.21 ± 0.54	0.305
HDL (mmol/L)	1.21 ± 0.38	1.21 ± 0.38	0.602	1.26 ± 0.31	0.475	1.31 ± 0.35	0.665	1.29 ± 0.36	0.248
Triglyceride (mmol/L)	1.67 ± 0.82	1.32 ± 0.73	**0.015**	1.37 ± 0.87	0.503	1.21 ± 0.61	0.576	1.38 ± 0.77	0.099

**Table 4:** Detailed statin dosage at 12-month follow-up visit.

Type of Statin	Dosage (mg)	Group 1 (*n* = 32)	Group 2 (*n* = 40)	*p*-Value	All (*n* = 72)
Atorvastatin	20	0 (0%)	1 (2.5%)	-	1 (1.4%)
	40	11 (34.4%)	16 (40%)	0.62	27 (37.5%)
	80	4 (12.5%)	5 (12.5%)	1.00	9 (12.5%)
Rosuvastatin	10	2 (6.3%)	2 (5%)	0.82	4 (5.6%)
	20	7 (21.9%)	10 (25%)	0.76	17 (23.6%)
	40	8 (25%)	6 (15%)	0.29	14 (19.4%)

## Data Availability

The data from the study are available upon reasonable request from the corresponding author.
